# Inequalities oral health between beneficiaries and non-beneficiaries of the Bolsa Família Program in Brazil: evidence from SB Brasil 2023

**DOI:** 10.1590/1980-549720260021.supl.1

**Published:** 2026-07-10

**Authors:** Marcos Pascoal Pattussi, Adriana Vieira Macedo Brugnoli, Francine Lorencetti da Silva Campioni, Renato Canevari Dutra da Silva

**Affiliations:** I Universidade do Vale do Rio dos Sinos, Graduate Program in Colletictive Health – São Leopoldo (RS), Brazil.; II Universidade de Rio Verde, School of Physiotherapy, Rio Verde Campus, Collective Health Research Group – Rio Verde (GO), Brazil.; III Universidade de Rio Verde, School of Dentistry, Rio Verde Campus – Rio Verde (GO), Brazil.; IV Universidade de Rio Verde, School of Dentistry, Rio Verde Campus, Collective Health Research Group – Rio Verde (GO), Brazil.

**Keywords:** Oral health, Health inequality, Bolsa Família program, Social determinants, Public policies

## Abstract

**Objective:**

To investigate inequalities in oral health between beneficiaries and non-beneficiaries of the Bolsa Família Program (PBF) in Brazil.

**Methods:**

This was a cross-sectional household study using data from the 2023 National Oral Health Survey (SB Brasil). Clinical indicators (caries, tooth loss, need for treatment and prosthesis) and subjective indicators (self-perception and dental pain) were evaluated. Associations between beneficiary status and outcomes were estimated using Poisson regression and multinomial logistic regression adjusted for sex, skin color, and dental visit in the last year.

**Results:**

Beneficiaries reported worse self-perception of oral health (PR 1.87; 95%CI 1.19–2.94 to PR 2.30; 95%CI 1.48–2.28) and a higher prevalence of caries, pain, and need for treatment. 12-year-old children reported more dental pain (PR 1.47; 95%CI 1.10–1.95). In adolescents, the greatest inequities occurred in caries severity (PR 2.03; 95%CI 1.45–2.83), need for urgent care (PR 2.29; 95%CI 1.54–3.39), and partial dentures (PR 2.66; 95%CI 1.65–4.29). Among adults, the need for partial (OR 2.11; 95%CI 1.56–2.85) and total (OR 3.38; 95%CI 1.65–3.42) prostheses was more frequent, a pattern that was maintained in the elderly (OR 1.61; 95%CI 1.05–2.47 and OR 2.06; 95%CI 1.36–3.13).

**Conclusion:**

Significant inequalities in oral health are observed between beneficiaries and non-beneficiaries of the Bolsa Família Program. Overcoming these inequities requires integration between income transfer policies, primary care, and specialized dental services, guided by the principles of equity and social justice.

## INTRODUCTION

Oral health is an integral part of overall health and plays a fundamental role in nutrition, speech, self-esteem, and psychological well-being^1^. Dental problems such as cavities, periodontal disease, and tooth loss contribute not only to pain and functional impairment, but are also associated with poorer quality of life, nutritional difficulties, and an increased risk of systemic diseases — including cardiovascular diseases, diabetes, metabolic syndrome, respiratory infections, and adverse pregnancy outcomes^1,2^.

Oral diseases remain among the most prevalent public health conditions and constitute important markers of social inequalities^3^, differences or inequalities that are unnecessary, avoidable, and ethically unjust^4^. It is estimated that oral problems and prosthetic needs affect populations in situations of vulnerability more intensely, increasing regional and socioeconomic disparities historically observed in Brazil^3,5^.

Individuals with lower income, lower educational attainment, or belonging to vulnerable groups show higher prevalence and severity of dental caries, periodontal disease, and tooth loss, as well as lower use of dental services^2,6,7^. Furthermore, critical and systematic reviews show that these inequities are universal and persistent over time, affecting children, adults, and older adults across different countries and health systems^8–10^.

In Brazil, systematic reviews and population-based cohort studies have consistently identified a strong association between lower socioeconomic status, measured by income, education, and economic class, and worse oral health outcomes, including a higher prevalence of untreated dental caries, edentulism, and poor self-rated oral health among Brazilian adults and children^11–13^.

In this scenario, initiatives were undertaken aiming to reduce or minimize socioeconomic and oral health inequalities in the country. The Bolsa Família Program (PBF), the Brazilian federal government's conditional cash transfer program, was implemented in 2003 with the objective of combating hunger and poverty in Brazil^14^. Smiling Brazil, established in 2004 by the Ministry of Health, promoted the incorporation of dentistry into primary care, the replacement of equipment, the formation of teams, as well as actions for the promotion, prevention, and recovery of oral health^15^. In this sense, one could expect these policies to contribute complementarily to reducing financial barriers and expanding equitable access to oral health services in Brazil. However, it has been argued that they lack coordination among themselves^16^. Despite the advances achieved with these public policies, inequalities in oral health and access to dental services^17^ still persist, disproportionately affecting the most vulnerable groups. Beneficiary children and schoolchildren show a higher prevalence and severity of dental caries, as well as lower access to dental services, compared to non-beneficiaries of the PBF^18^.

However, there is a shortage of comprehensive national studies comparing multiple oral health indicators between beneficiaries and non-beneficiaries of the Bolsa Família Program across different age groups. In this regard, the objective of the present study is to estimate the prevalences and compare the differences in oral health conditions between beneficiaries and non-beneficiaries of the Bolsa Família Program among children, adolescents, adults, and older adults, based on data representative of the national population.

## METHODS

### Study design and data source

This cross-sectional study used data from the National Oral Health Survey (SB Brasil) 2023, a household survey conducted throughout the national territory, whose detailed methodology is described in the final research report^5^. The study was designed to estimate oral health indicators by age groups and 53 geographic domains (Brazil, Federative Units, Federal District, and state capitals). The clinical variables were collected by trained and calibrated examiners and recorded in an electronic system, using the criteria and recommendations of the World Health Organization (WHO).

### Population and sampling

The population included residents of urban and rural households in Brazil. Sampling was probabilistic, complex, and multi-stage cluster sampling. Individuals were examined at the following ages: 5 years (deciduous dentition); 12 years (permanent dentition); 15–19 years (adolescents); 35–44 years (adults); and 65–74 years (elderly), allowing for the estimation of internationally comparable indicators and the assessment of the life course of oral health. Results for the 5-year-old group are presented in supplementary tables. The sample size was calculated considering expected prevalences of caries and periodontal disease, a precision of 5%, a design effect (deff=2), and a 20% increase for losses, totaling approximately 50,000 individuals across all regions of the country.

### Training and calibration

The examiners were previously trained dental surgeons who underwent theoretical and practical calibration. Intra- and inter-examiner agreement was assessed using the Kappa coefficient (minimum acceptable ≥0.65), ensuring the standardization of diagnostic criteria for all oral conditions investigated. The record keepers also underwent specific training to guarantee the reliability of the records.

### Data collection and quality control

Data collection occurred through home visits and consisted of: a structured interview, applied to participants or legal guardians (in the case of minors), covering sociodemographic, behavioral, and dental service utilization data; in addition to clinical examinations performed with plane mirrors and WHO probes, under natural light, in a well-ventilated and illuminated area of the home, following biosafety standards. The following conditions were evaluated: dental caries (dmft/DMFT indices), clinical consequences of untread dental caries (PUFA), treatment needs, periodontal health (CPI), edentulism, use and need for prostheses, malocclusion (DAI), and dental trauma.

### Variables

#### Outcomes

Clinical and subjective indicators of oral health were analyzed in the investigated age groups (children, adolescents, adults, and the elderly). Subjective variables included: toothache in the last six months (yes/no); perceived urgency in dental treatment (unnecessary; preventive or routine; elective treatment; need for immediate treatment); and self-perception of oral health (fair/good/very good; poor/very poor). The decision to combine the categories "fair," "very good," and "good" is consistent with the epidemiological literature and aimed to distinguish individuals who clearly perceive their oral health as problematic from those who do not report relevant problems, since the "fair" category tends to represent an intermediate or neutral assessment, while "poor" and "very poor" explicitly indicate the presence of problems or dissatisfaction with oral health.

The clinical variables comprised the assessment of dental caries, measured by the dmft (primary dentition) and DMFT (permanent dentition) indices, including the number of decayed, restored, and missing teeth. The presence of untreated caries was categorized as "none" or "one or more teeth." The clinical consequences of caries were measured by the PUFA index (PUFA=0; PUFA≥1). In adults and the elderly, the presence of root caries (yes/no) and the occurrence of permanently extracted teeth (yes/no) were also assessed.

The need for dental treatment was classified as restoration, pulp treatment, or extraction (none/≥1 tooth in younger age groups; yes/no in adults and the elderly). Periodontal conditions were also included among adults and the elderly, assessed by the presence of any periodontal attachment loss and periodontal pockets (yes; no). Edentulism was defined as the total absence of natural teeth (dentate; edentulous), and the need for dental prostheses was categorized as not needed, needs partial prosthesis, or needs complete prosthesis. With the exception of some categorizations, the indicators already calculated in the database provided by the Ministry of Health were used.

#### Main Exhibition

The primary exposure was assessed by asking whether any resident had received income from social assistance benefits from the Bolsa Família Program in the last year (no; yes). Participants who did not know or did not answer this question were excluded.

#### Other independent variables

The independent variables included sociodemographic, contextual, and dental service use characteristics. Contextual variables encompassed geographic region of Brazil (North; Northeast; Southeast; South; Midwest) and type of city (capital; interior). Individual characteristics included sex (male; female) and race/skin color (white; black; yellow; brown; indigenous).

Socioeconomic status was assessed through monthly family income, per capita income, and number of people per room in the household (all in continuous values); in addition to the mother's education level and the participant's education level, classified into eight categories: not studying, completed Youth and Adult Education (EJA), incomplete/complete Elementary School (ES), incomplete/complete High School (HS), and incomplete/complete Higher Education (HE).

The use of dental services was analyzed according to the last visit to the dentist (never; up to 1 year; more than 1 to 2 years; more than 2 to 3 years; more than 3 years) and the main reason for the visit (never been to the dentist; cleaning, prevention or check-up; toothache; extraction; dental treatment; gum problem; treatment of oral wound; dental implant; placement/maintenance of orthodontic appliance; placement/maintenance of prosthesis or denture; other).

### Data entry and analysis

The data were double-entered with automatic validation to ensure consistency and quality. The analyses considered the complex sampling design, incorporating weights, strata, and clusters, according to the technical specifications of SB Brasil 2023 and using the svy command in *Stata* 15.1 software.

Initially, frequencies, means, and prevalences of the main oral health problems were calculated, stratified according to whether the beneficiaries were or were not beneficiaries of the Bolsa Família Program (PBF) and the age groups surveyed. Subsequently, adjusted Prevalence Ratios (PR) were estimated for each specific oral health outcome using multiple Poisson regression models and Wald tests for linear trend and heterogeneity of proportions, with the receipt of the benefit as the main exposure variable. For the outcome of need for prostheses, among adults and the elderly, adjusted Odds Ratios (OR) were estimated using multiple multinomial logistic regression, since it is a nominal categorical outcome with more than two categories without natural ordering. The models were adjusted for potential confounding factors, identified based on the literature and statistical significance (p<0.05). To avoid collinearity with the main exposure, socioeconomic variables were not included in the multivariate models. Thus, the effect of being a beneficiary of the PBF was adjusted for sex, skin color, and having a dental appointment in the last 12 months.

### Ethical aspects

The study was approved by the National Research Ethics Committee (Conep – National Research Ethics Committee/CNS – National Health Council/MS – Ministry of Health, CAAE 34497120.6.3001.0008, opinion number 4.823.054), and all participants signed the Informed Consent Form (TCLE) or, in the case of minors, the Assent and Consent Form from their guardians. The research followed the ethical principles of the Declaration of Helsinki.

#### Data availability statement:

The entire dataset supporting the results of this study is available upon request from the corresponding author. The dataset is not publicly available due to database integration performed by the authors.

## RESULTS

In total, 40,720 individuals participated in SB Brasil 2023. Of these, 3,200 did not know or did not answer whether they had received income from the Bolsa Família Program and, therefore, were excluded from the analyses. Thus, the analyses were conducted with 37,520 participants, distributed among the different age groups. Of the total analyzed, 11,757 (31.4%) were beneficiaries of the Bolsa Família Program, and 25,763 (68.6%) belonged to the general non-beneficiary population.

Beneficiaries of the aforementioned program presented a distinct profile compared to the general population. There was a greater concentration in the Northeast and Southeast regions, while in the general population the Southeast and Northeast predominated. From a socioeconomic point of view, as expected, the beneficiaries exhibited worse income and education conditions, with significantly lower family and per capita income and a higher proportion of participants with incomplete primary education. Regarding the use of dental services, PBF beneficiaries also mostly used them for cleaning, prevention, or check-ups; however, the proportion of use due to toothache among beneficiaries was higher than in the general population (Table 1).

**Table 1:** Demographic, socioeconomic, and dental service utilization characteristics according to beneficiary status in the Bolsa Família Program. National Oral Health Survey (SB Brasil), 2023.

Variables		Bolsa Família			General population		
		n	%	95%CI	n	%	95%CI
Regions of Brazil							
	North	3,814	15.8	12.7–19.6	5,523	6.4	5.1–8.0
	North East	5,294	44.4	39.0–49.9	8,216	20.6	17.6–23.9
	Southeast	791	25.1	20.6–30.3	3,712	47.4	41.2–53.7
	South	697	6.6	4.9–8.7	4,362	17.4	14.6–20.7
	Central-West	1,161	7.9	5.1–12.0	3,950	7.9	6.0–10.4
City type							
	Capital	8,452	22.7	19.9–25.7	18,914	23.0	20.0–26.4
	Interior	3,305	77.3	74.2–80.1	6,849	76.9	73.5–79.9
Age range (years)							
	5	3,051	7.29	6.5–8.2	3,433	2.89	2.5–3.4
	12	2,487	6.5	5.7–7.4	3,499	3.3	2.8–3.8
	15–19	2,583	30.7	28.1–33.5	4,791	18.6	16.7–20.6
	35–44	2,481	47.7	44.6–50.9	6,006	50.0	47.2–52.8
	65–74	1,155	7.6	6.5–8.9	8,034	25.1	23.2–27.0
Sex							
	Male	3,896	35.7	33.1–38.3	10,539	40.9	39.2–42.5
	Female	7,861	64.3	61.6–66.8	15,223	59.0	57.4–60.7
Race/color							
	White	2,556	24.4	21.3–27.8	12,511	48.7	44.9–53.2
	Black	1,692	17.3	13.7–21.5	2,972	11.0	9.9–12.3
	Yellow	165	1.6	1.0–2.7	283	1.0	0.7–1.4
	Brown	7,150	55.7	50.7–58.9	8,764	38.0	34.7–42.3
	Indigenous	58	0.6	0.3–1.2	80	0.3	0.1–0.5
	Could not inform.	136	1.3	0.7–2.4	1,153	1.2	0.7–1.9
Family income							
		8,727	1,626*	1,502–1,749	18,907	3569.1*	3,310–3,828
Per capita income							
		8,705	422.2*	389–456	18,879	1,197*	1,103–1,290
People/room							
		11,656	1.9*	1.9–2.1	25,539	1.4*	1.3–1.5
Participant's education level							
	Without instruction	298	2.8	2.1–3.9	1,045	3.5	2.8–4.2
	Incomplete primary school	4,112	32.6	29.2–36.3	7,792	22.0	19.8–24.4
	PrimarySchoolComplete/Secondary Incomplete	2,321	34.7	31.7–37.8	4,992	24.9	22.4–27.6
	Complete secondary education or higher	1,865	28.6	25.4–32.0	8,240	48.1	45.2–51.0
	No response	110	1,3	0,7–2,3	261	1,5	0,8–2,7
Last dentist appointment							
	Never	2.147	7,6	6,1–9,4	1.838	2,3	1,8–2,8
	Up to one year	4.771	43,7	39,9–47,7	12.389	48,1	45,0–51,2
	More than 1 to 2 years	1.924	18,6	16,0–21,7	4.436	17,6	16,1–19,1
	More than 2 to 3 years	1.036	9,8	8,3–11,7	2.630	11,6	10,2–13,2
	More than 3 years	1.374	14,7	11,9–18,1	3.955	15,7	13,8–17,8
	I don't know	505	5,4	3,6–7,9	1.141	4,7	3,1–7,0
Main reason for the consultation							
	Never been to the dentist	2.147	7,5	6,1–9,4	1.838	2,3	1,8–2,8
	Cleaning, prevention or inspection	3.318	25,9	23,5–28,6	826	30,7	27,8–33,5
	Toothache	1.811	17,3	15,2–19,8	2.763	12,9	10,9–15,0
	Extraction	1.303	13,2	11,4–15,5	2.761	8,9	7,7–10,1
	Dental treatment	1.377	16,3	13,8–19,3	3.501	17,5	15,7–19,4
	Gum problem	58	0,5	0,3–1,0	209	0,8	0,5–1,1
	Mouth sore	18	0,1	0,2–0,1	55	0,2	0,1–0,5
	Dental implant	13	0,1	0,0–0,3	324	1,8	1,2–2,7
	Orthodontic consultation	461	6,2	5,0–7,8	1.434	6,6	5,5–7,7
	Prosthesis consultation	382	3,6	2,8–4,7	2.599	9,9	8,6–12,1
	Others	207	2,2	1,5–3,4	521	2,6	1,9–3,4

* Means. CI: confidence interval.

Among the participating children and adolescents, significant differences were observed in oral health conditions between beneficiaries and non-beneficiaries. In all age groups analyzed (12 and 15–19 years), beneficiaries presented a higher prevalence of toothache, worse self-perception of oral health, and greater needs for dental treatment, especially immediate treatment. In addition, the dental caries experience (DMFT) and complications resulting from dental caries (PUFA) indices were consistently higher among beneficiaries compared to non-beneficiaries (Table 2).

**Table 2:** Oral health conditions in children and adolescents according to their eligibility for the Bolsa Família Program. National Oral Health Survey (SB Brasil) 2023.

Variables		12 years						15 to 19 years old					
		Bolsa Família			General population			Bolsa Família			General population		
		n	%	95%CI	n	%	95%CI	n	%	95%CI	n	%	95%CI
Toothache													
	No	1,911	77.7	73.3–81.5	2,926	85.9	82.5–88.7	1,926	74.7	70.4–8.6	3,820	80.5	77.5–83.3
	Yes	563	22.4	18.5–26.7	554	14.1	11.3–17.4	642	25.3	21.4–29.5	945	19.5	16.7–2.52
Self-perception in oral health													
	Good/very good	2,263	93.7	91.3–95.5	3,297	97.2	96.1–97.9	2,354	91.1	87.5–93.7	4,509	96.4	94.4–97.7
	Average/bad/very bad	171	6.3	4.5–8.7	144	2.8	2.0–3.8	193	8.9	6.2–12.5	218	3.6	2.2–5.5
Urgent need													
	No need	455	22.6	18.6–27.1	997	37.1	32.4–41.9	451	19.3	15.1–24.1	1,319	34.1	26.9–41.9
	Preventive treatment	840	31.9	27.9–36.0	1,128	27.7	24.6–30.9	774	27.7	22.6–33.2	1,382	28.7	24.2–33.5
	Tratamento eletivo	943	37.4	33.2–41.7	1,172	30.8	25.6–36.4	1,031	39.2	33.6–45.1	1,709	31.8	27.7–36.2
	Need for immediate treatment.	245	8.2	6.0–10.9	201	4.4	3.2–5.9	323	13.9	10.6–17.8	373	5.4	4.1–7.1
DMFT*													
		2,483	1.8	1.6–2.1	3,498	1.5	1.2–1.7	2,579	3.7	3.2–4.2	4,783	3.1	2.6–3.5
Caries*													
		2,483	1.3	1.1–1.4	3,498	0.9	0.7–1.0	2,579	2.2	1.8–2.5	4,783	1.2	0.9–1.4
Restored*													
		2,483	0.5	0.3–0.6	3,498	0.6	0.4–0.7	2,579	1.0	0.7–1.2	4,783	1.5	1.2–1.7
Lost*													
		2,483	0.1	0.05–0.1	3,498	0.1	0.03–0.07	2,579	0.6	0.3–0.7	4,783	0.5	0.3–0.6
Untreated tooth decay													
	C=0	1,350	57.2	53.2–61.1	2,327	68.3	62.5–73.5	1,284	44.8	38.9–50.8	2,946	62.5	58.1–66.7
	C≥1	1,133	42.8	38.8–46.8	1,171	31.7	26.4–37.4	1,295	55.2	49.2–61.1	1,837	37.5	33.2–41.9
Experience of cavities													
	DMFT = 0	1,043	46.2	41.9–50.4	1,833	53.9	48.3–59.4	819	28.5	23.6–33.8	1,776	36.9	32.4–41.6
	CPO-D≥1	1,440	53.9	49.5–58.1	1,665	46.1	40.5–51.6	1,760	71.5	66.2–76.3	3,007	63.1	58.3–67.5
PUFA prevalence													
	PUFA=0	2,176	88.1	85.0–90.6	3,222	93.9	92.1–95.3	2,121	79.6	73.4–84.7	4,258	91.4	88.9–93.2
	PUFA≥1	307	11.9	9.3–14.9	276	6.1	4.7–7.8	458	20.4	15.3–26.6	525	8.6	6.7–11.0
Need for treatment													
	No teeth	1,382	58.6	54.5–62.4	2,356	70.3	65.9–74.3	1,303	47.5	42.1–53.0	2,961	63.4	58.4–68.1
	≥1 tooth	1,101	41.4	37.5–45.4	1,142	29.7	25.6–34.1	1,276	52.5	46.9–57.9	1,822	36.6	31.8–41.5
Need for restoration													
	None	1,473	62.2	58.2–66.0	2,437	71.7	67.4–75.7	1,412	52.0	46.6–57.3	3,126	66.1	61.4–70.5
	≥1 tooth	1,010	37.8	33.9–41.8	1,061	28.3	24.2–32.6	1,167	48.0	42.6–53.3	1,657	33.9	29.4–38.5
Need for pulp treatment													
	No teeth	2,262	91.8	89.1–93.8	3,305	95.5	94.0–96.6	2,289	88.2	84.8–90.9	4,438	94.3	92.7–95.6
	≥1 tooth	221	8.2	6.15–10.9	193	4.5	3.3–5.9	290	11.8	9.0–15.1	345	5.7	4.3–7.3
Need for extraction													
	No teeth	2,374	96.0	94.3–97.2	3,411	98.4	97.6–98.9	2,339	88.6	84.7–91.5	4,508	95.8	94.3–96.9
	≥1 tooth	109	4.0	2.7–5.7	87	1.6	1.0–2.3	240	11.4	8.5–15.2	275	4.2	3.1–5.7
Prevalence extracted													
	E=0	2,320	93.9	92.0–95.3	3,355	96.6	95.2–97.5	2,110	80.9	76.8–84.3	4,207	89.4	86.9–91.3
	E≥1	163	6.1	4.6–7.9	143	3.4	2.4–4.7	469	19.1	15.7–23.1	576	10.7	8.6–13.0

* Means. CI: confidence interval.

With regard to dental trauma, malocclusions, and the presence of bleeding and calculus, no significant differences were observed between beneficiaries and non-beneficiaries of the Bolsa Família program (data not shown).

Regarding adults and the elderly, a consistent pattern of poorer oral health conditions was observed among beneficiaries compared to non-beneficiaries. In both groups, beneficiaries presented worse self-perception of oral health, a higher incidence of toothache, and greater treatment needs, especially immediate ones. Furthermore, a greater experience and severity of caries was observed, with a predominance of tooth loss and a lower proportion of restored teeth among beneficiaries. It is also noteworthy that the demand for rehabilitative treatment, such as the need for partial or complete dentures, was approximately twice as frequent in beneficiaries than in the general population (Table 3).

**Table 3:** Oral health conditions in adults and the elderly according to beneficiary status of the Bolsa Família Program. National Oral Health Survey (SB Brasil) 2023.

Variables		35 to 44 years old						65 to 74 years old					
		Bolsa Família			General population			Bolsa Família			General population		
		n	%	95%CI	n	%	95%CI	n	%	95%CI	n	%	95%CI
Toothache													
	No	1,654	70.4	65.2–75.2	4,603	77.4	73.8–80.7	928	89.4	84.9–92.6	6,676	87.7	85.2–89.8
	Yes	811	29.5	24.7–34.8	1,347	22.5	19.2–26.2	149	10.6	7.3–15.0	956	12.3	10.2–14.7
Self-perception in oral health													
	Good/very good	1,857	75.3	70.4–79.7	5,146	87.3	84.7–89.6	856	74.6	66.8–81.1	6,476	86.48	84.4–88.3
	Average/bad/very bad	592	24.6	20.2–29.5	767	12.6	10.3–15.2	247	25.4	18.9–33.2	1263	13.52	11.6–15.6
Urgent need													
	No need	242	12.4	9.3–16.3	1,242	21.3	17.1–26.1	265	28.2	21.5–36.1	2,045	29.1	25.2–33.3
	Preventive treatment	393	19.9	15.3–25.5	1,242	22.0	18.8–25.5	155	9.7	7.0–13.3	1,270	16.9	14.4–19.9
	Elective treatment	1,365	51.6	46.3–56.9	2,896	48.2	42.9–53.5	578	52.6	44.8–60.3	4,044	46.4	42.2–50.6
	Need for immediate treatment.	475	15.9	12.7–19.9	600	8.4	6.6–10.6	156	9.3	6.2–13.8	654	7.4	5.8–9.4
DMFT*													
		2,475	11.8	10.8–12.8	5,980	10.5	9.9–11.1	1,154	25.2	23.8–26.5	8,014	23.3	22.6–24.0
Caries*													
		2,475	2.9	2.4–3.4	5,980	1.6	1.4–1.8	1,154	0.9	0.7–1.2	8,014	0.9	0.7–1.1
Restored*													
		2,475	3.9	3.4–4.4	5,980	5.9	5.5–6.3	1,154	1.2	0.9–1.5	8,014	3.0	2.4–3.6
Lost*													
		2,475	4.9	4.2–5.7	5,980	3.0	2.6–3.5	1,154	22.9	21.4–24.5	8,014	19.3	18.2–20.4
PUFA prevalence													
	PUFA=0	1,755	75	70.3–79.1	4,939	83.4	80.6–85.9	982	89.1	85.7–91.8	7,137	90.0	88.1–91.6
	PUFA≥1	720	25	20.8–29.6	1,041	16.6	14.1–19.4	172	10.8	8.1–14.2	878	9.9	8.3–11.8
Root caries													
	None tooth	1,773	70.2	64.5–75.3	4,922	82.3	78.5–85.5	924	81.4	75.1–86.4	6,662	83.0	79.7–85.9
	≥1 tooth	702	29.8	24.6–35.5	1,058	17.7	14.5–21.4	230	18.5	13.5–24.8	1,353	16.96	14.08–20.3
Need for treatment													
	No teeth	769	32.1	26.5–38.2	2,986	49.7	45.5–53.8	755	67.0	59.6–73.8	5,424	69.2	65.5–72.6
	≥1 tooth	1,706	67.8	61.8–73.4	2,994	50.3	46.1–54.4	399	32.9	26.2–40.4	2,591	30.7	27.3–34.4
Need for restoration													
	No teeth	948	37.7	31.9–43.8	3,349	55.8	52.1–59.5	845	75.6	69.2–81.0	5,962	74.1	70.5–77.4
	≥1 tooth	1,527	62.3	56.1–68.0	2,631	44.1	40.5–47.9	309	24.3	18.9–30.7	2,053	25.9	22.5–29.5
Need for pulp treatment													
	No teeth	2,166	88.3	84.9–91.0	5,497	91.5	89.1–93.4	1,110	96.7	94.6–98.0	7,755	96.8	95.3–97.9
	≥1 tooth	309	11.7	8.9–15.1	483	8.5	6.5–10.8	44	3.2	1.9–5.4	260	3.1	2.1–4.6
Need for extraction													
	No teeth	1,875	79.3	75.4–82.7	5,215	87.9	85.4–90.1	989	87.9	81.9–92.1	7,098	90.1	88.2–91.7
	≥1 tooth	600	20.6	17.3–24.5	765	12.0	9.9–14.5	165	12.1	7.87–18.1	917	9.8	8.27–11.7
Extracted													
	No teeth	748	28.9	23.3–35.3	2,623	43.3	38.0–48.8	78	2.7	1.6–4.5	732	10.8	8.6–13.5
	≥1 tooth	1,727	71.0	64.7–76.6	3,357	56.6	51.2–61.9	1,076	97.2	95.5–98.3	7,282	89.2	86.4–91.4
Insertion loss													
	No	1,541	83.7	78.8–87.6	4,306	84.3	78.7–88.7	83	69.9	54.5–81.8	773	59.6	49.7–68.8
	Yes	289	16.3	12.3–21.2	674	15.6	11.3–21.2	41	30.1	18.1–45.4	481	40.3	31.1–50.2
Bolsa													
	No	1,529	83.8	78.3–88.1	4,372	85.8	82.4–88. 6	93	76.3	61.0–86.9	998	77.2	69.9–83.2
	Yes	301	16.1	11.8–21.6	608	14.1	11.3–17.6	31	23.6	13.0–38.9	258	22.7	16.7–30.1
Edentulism													
	No	2,434	98.6	96.4–99.4	5,912	99.3	98.8–99.6	683	53.6	45.8–61.3	5,270	65.4	61.9–68.7
	Yes	41	1.3	0.5–3.5	68	0.6	0.3–1.1	471	46.3	38.7–54.1	2,744	34.5	31.2–38.0
Need for prosthesis													
	No need	743	30.4	25.1–36.2	2,967	50.8	45.0–56.5	198	13.0	9.7–17.3	1,947	26.2	23.1–29.6
	Partial need	1,622	65.3	59.4–70.7	2,877	47.0	41.1–53.0	393	31.6	25.8–38.0	2,875	36.3	33.6–39.1
	Total needs	108	4.3	2.7–6.8	135	2.1	1.3–3.4	562	55.3	48.7–61.8	3,190	37.4	33.9–41.1

* Averages.

CI: confidence interval.

After adjusting for sex, skin color, and dental consultation, it was observed that children and adolescents benefiting from the Bolsa Família Program presented higher prevalences of dental pain, negative self-perception of oral health, and need for dental treatment when compared to non-beneficiaries. The adjusted prevalence ratios indicated a significant association between receiving the benefit and a higher occurrence of untreated caries, caries-related complications (PUFA), and need for immediate treatment (Table 4).

**Table 4:** Association between Bolsa Família Program beneficiary status (versus non-beneficiary) and different oral health outcomes according to age. National Oral Health Survey (SB Brasil) 2023.

Variable	12 years		15 to 19 years old		35 to 44 years old		65 to 74 years old	
	PR	95%CI	PR	95%CI	PR	95%CI	PR	95%CI
Self-perception of oral health	1.87	1.19–2.94	2.11	1.12–3.97	1.84	1.48–2.28	1.67	1.25–2.22
Toothache	**1.47**	**1.10–1.95**	1.21	0.98–1.50	**1.29**	**1.08–1.55**	0.96	0.65–1.43
DMFT	1.15	0.94–1.41	1.11	0.96–1.31	**1.11**	**1.02–1.21**	1.02	0.73–1.46
Spoiled	1.28	1.04–1.59	**1.64**	**1.34–2.01**	**1.77**	**1.50–2.09**	1.08	1.00 –1.17
Lost	1.22	0.75–1.99	1.15	0.67–1.97	**1.54**	**1.25–1.90**	**1.06**	**1.03–1.09**
Restored	0.92	0.67–1.25	**0.66**	**0.50–0.86**	**0.69**	**0.59–0.80**	0.60	0.43–0.83
Caries ≥1	**1.22**	**1.01–1.47**	**1.35**	**1.22–1.50**	**1.35**	**1.22–1.49**	1.09	085–1.40
Extracted ≥1	1.41	0.95–2.09	**1.63**	**1.19–2.24**	**1.19**	**1.07–1.32**	1.06	1.03–1.09
PUFA – permanent	**1.67**	**1.16–2.38**	**2.03**	**1.45–2.83**	**1.49**	**1.18–1.89**	1.01	0.72–1.40
Need for treatment	**1.28**	**1.10–1.50**	**1.32**	**1.14–1.53**	**1.31**	**1.19–1.45**	1.06	0.82–1.36
Need for restoration	**1.24**	**1.06–1.46**	**1.31**	**1.12–1.53**	**1.38**	**1.24–1.54**	0.95	0.72–1.25
Need for endodontics	**1.58**	**1.03–2.43**	**1.81**	**1.29–2.54**	1.35	0.95–1.91	0.95	0.47–1.89
Need for extraction	2.32	**1.24–4.33**	**2.11**	**1.40–3.18**	**1.79**	**1.40–2.29**	1.05	0.66–1.70
Urgent need	**1.65**	**1.05–2.56**	**2.29**	**1.54–3.39**	**1.91**	**1.42–2.57**	1.05	0.65–1.73
Need for a partial prosthesis*			2.66	1.65–4.29	2.11	1.56–2.85	1.61	1.05–2.47
Need for a complete denture*			6.64	0.48–91.8	**3.38**	**1.65–3.42**	**2.06**	**1.36–3.13**
Root caries ≥1					**1.61**	**1.26–2.06**	1.08	0.75–1.58
Insertion loss					1.01	0.66–1.54	0.70	0.44–1.10
Periodontal pocket					1.16	0.79–1.71	0.89	0.50–1.60
Edentulism					2.28	0.71–7.31	1.16	0.97–1.40

Note: Values in bold are statistically significant (p<0.005).

* Odds Ratio (OR). Adjusted for sex, skin color, and dental visit in the last 12 months.

PR: prevalence ratio; CI: confidence interval.

Among adults, the observed pattern was similar to that of younger age groups in the adjusted analysis, indicating the persistence of inequities in oral health. Beneficiaries of the Bolsa Família Program (PBF) presented higher prevalences of poor self-perception of oral health, pain, worse clinical indicators, and a greater need for treatment when compared to non-beneficiaries. Regarding prosthetic rehabilitation, it is noteworthy that the need for complete and partial dentures was significantly higher among beneficiaries compared to non-beneficiaries, both among adults and the elderly (Table 4).

## DISCUSSION

The results of this study highlight striking inequities in oral health between beneficiaries and non-beneficiaries of the Bolsa Família Program across all age groups analyzed. Oral health inequities refer to unjust inequalities or differences among different population groups regarding oral health and access to dental services, relating to racial, social, economic, and territorial factors^19,20^. Children, adolescents, adults, and older adults linked to the program presented worse clinical and subjective indicators, a higher prevalence of dental pain, poorer self-perception of oral health, greater experience and severity of caries, as well as a greater need for dental treatment, especially of an immediate nature^21^.

These inequities reflect a pattern widely described in the literature, according to which socioeconomic position, expressed through income, education, occupation, and place of residence, is strongly associated with unfavorable oral health conditions^6,7^. Systematic reviews demonstrate that individuals living in poverty present higher rates of dental caries, tooth loss, periodontitis, and lower utilization of dental services. These inequities result from social determinants, including limited access to care, low health literacy, and cultural and geographic barriers to dental care^22–24^.

Although oral diseases are largely preventable, they remain at alarming levels worldwide because they reflect structural inequalities, constituting a global "oral injustice"^2^.

Even in countries with universal healthcare systems, such as Brazil, inequities persist. National studies show that, despite the expansion of primary care and the free provision of dental services through the Unified Health System (SUS), individuals with lower income and education levels continue to use preventive and rehabilitative services less frequently, seeking care predominantly in situations of pain or urgency. This "inequity inertia" reflects the reproduction of historical disadvantages and geographic and symbolic barriers that have still not been overcome^21,22^.

The PBF, as a conditional cash transfer policy, is a well-established instrument for poverty mitigation. Evidence shows its positive effects on outcomes such as the reduction of infant mortality and low birth weight. However, its direct impact on the intermediate determinants of oral health and access to dental services is still limited^24,25^. Structural obstacles persist, such as the distance to health units, the shortage of teams in vulnerable areas, and socioeconomic barriers that limit the regular use of dental services and encourage seeking care only for emergencies^26^.

National studies and systematic reviews^18,27,28^ point to a higher prevalence of dental caries and lower use of dental services among beneficiaries of income transfer programs compared to the general population, indicating that income transfer, although essential for reducing economic vulnerabilities, is not sufficient to eliminate structural barriers or guarantee equitable access. Therefore, the need to integrate income transfer policies such as Bolsa Família with Basic Health Units and the oral health teams of the Family Health Strategy becomes evident. Measures such as incentives for preventive visits, prioritization of dental care for beneficiaries, and intersectoral actions involving health, education, sanitation, and social assistance are needed. This converges with international recommendations on poverty reduction and oral health policies^2,29^.

It is likely that Bolsa Família provides benefits to oral health among the most vulnerable populations, especially when received over prolonged periods. A study in Fortaleza demonstrated an 87% reduction in the probability of dental caries in children who had received the benefit for at least two years^28^, suggesting a protective effect resulting from improved socioeconomic conditions. However, when the comparison is extended to the general population, inequalities remain evident, indicating that the program contributes to mitigating extreme inequities, but its scope is insufficient to eliminate the structural effects of poverty on health outcomes.

Among adults and older adults, the highest prevalences of pain, tooth loss, and need for prostheses reflect the concept of "accumulated disadvantage," in which social vulnerability limits restorative treatments and perpetuates mutilating practices^2^.

With the exception of the need for complete and partial dentures, an apparently smaller disparity was observed between beneficiaries and non-beneficiaries in the other oral health indicators among older adults. This finding may be explained by the high prevalence of edentulism in both groups, which tends to reduce relative differences. Such a pattern reflects the historical legacy of a predominantly tooth extraction-based oral health care model that preceded the implementation of Brasil Sorridente and contributed to widespread tooth loss across different social strata^30^.

The greater tooth loss and consequent need for prostheses among adult and elderly beneficiaries of the PBF reveal barriers in access to rehabilitative and emergency care and, furthermore, an accumulation of disadvantages throughout life. In this regard, it is essential to strengthen the integration of the program with the Dental Specialty Centers (CEO) of the Brazilian Unified Health System (SUS).

The status of being a PBF beneficiary remained associated with worse oral health outcomes, regardless of the adjustment variables included in the models^31,32^. Although the Bolsa Família Program contributes to reducing food insecurity and improving living conditions, its effectiveness, especially regarding oral health, depends on coordination with health policies, particularly primary care^26^. The persistence of a care model centered on curative procedures limits progress toward equity and reinforces the need for integration between social protection and oral health promotion^22,33,34^.

Oral health surveillance constitutes a strategic axis for monitoring inequities and evaluating the impact of public policies. Its articulation with the qualification and expansion of oral health teams and specialty centers represents a concrete alternative for reducing these inequities, since the presence of these teams is associated with an increase in preventive, restorative, and rehabilitative procedures^33^.

This study presents limitations inherent to its cross-sectional design, precluding causal inferences. There is also the possibility of residual bias resulting from the absence of relevant contextual variables, such as water fluoridation and coverage by family oral health teams, as well as individual factors, such as duration of participation in the program and behavioral habits related to oral health. In addition, the relevance of the PBF was not assessed in comparisons across different levels of vulnerability, especially among groups living in extreme poverty, which could deepen the understanding of its effects on reducing inequities. The findings should be interpreted in light of the possibility of residual confounding, since key socioeconomic variables, such as income and education, although strongly related to eligibility for the PBF, were not included in the adjusted models. Thus, the results reflect inequalities associated with being a beneficiary of the program. On the other hand, the large national sample and the methodological standardization in data collection confer robustness and high external validity to the findings.

The results confirm profound oral health inequities between beneficiaries and non-beneficiaries of the Bolsa Família Program, highlighting the persistent effect of social vulnerability on oral health outcomes. Addressing these inequities requires effective coordination between social protection and health policies, especially with primary care and specialized dental services, guiding public actions by the principle of social justice and the universal right to oral health.
