# Supplementary material for: Inequalities oral health between beneficiaries and non-beneficiaries of the Bolsa Família Program in Brazil: evidence from SB Brasil 2023
Source: Rev Bras Epidemiol. 2026 Jul 10;29(Suppl 1):e260021supl1. doi: 10.1590/1980-549720260021.supl.1 (PMC13360977; doi:10.1590/1980-549720260021.supl.1)
Supplement: Translation

# Translation: Iniquidades em saúde bucal entre beneficiários e não beneficiários do Programa Bolsa Família no Brasil: evidências do SB Brasil 2023

**DOI:** 10.1590/1980-549720260021.supl.1.2

**Authors:** Marcos Pascoal Pattussi, Adriana Vieira Macedo Brugnoli, Francine Lorencetti da Silva Campioni, Renato Canevari Dutra da Silva

**Affiliations:** I Universidade do Vale do Rio dos Sinos, Programa de Pós-Graduação em Saúde Coletiva – São Leopoldo (RS), Brasil.; II Universidade de Rio Verde, Faculdade de Fisioterapia do Campus Rio Verde, Grupo de Pesquisa em Saúde Coletiva – Rio Verde (GO), Brasil.; III Universidade de Rio Verde, Faculdade de Odontologia do Campus Rio Verde – Rio Verde (GO), Brasil.; IV Universidade de Rio Verde, Faculdade de Odontologia do Campus Rio Verde, Grupo de Pesquisa em Saúde Coletiva – Rio Verde (GO), Brasil.

**Keywords:** Saúde bucal, Iniquidade em saúde, Programa bolsa família, Determinantes sociais, Políticas públicas

## Abstract

**Objetivo:**

Investigar iniquidades em saúde bucal entre beneficiários e não beneficiários do Programa Bolsa Família (PBF) no Brasil.

**Métodos:**

Estudo transversal domiciliar com dados da Pesquisa Nacional de Saúde Bucal (SB Brasil) 2023. Avaliaram-se indicadores clínicos (cárie, perda dentária, necessidade de tratamento e prótese) e subjetivos (autopercepção e dor dentária). As associações entre condição de beneficiário e desfechos foram estimadas por regressão de Poisson e logística multinomial ajustada por sexo, cor da pele e consulta odontológica no último ano.

**Resultados:**

Beneficiários relataram pior autopercepção de saúde bucal (RP 1,87; IC95% 1,19–2,94 a RP 2,30; IC95% 1,48–2,28) e maior prevalência de cáries, dor e necessidade de tratamento. Crianças de 12 anos apresentaram mais dor dentária (RP 1,47; IC95% 1,10–1,95). Em adolescentes, as maiores iniquidades ocorreram na severidade de cárie (RP 2,03; IC95% 1,45–2,83), necessidade de urgência (RP 2,29; IC95% 1,54–3,39) e prótese parcial (RP 2,66; IC95% 1,65–4,29). Entre adultos, a necessidade de prótese parcial (OR 2,11; IC95% 1,56–2,85) e total (OR 3,38; IC95% 1,65–3,42) foi mais frequente, padrão que se manteve em idosos (OR 1,61; IC95% 1,05–2,47 e OR 2,06; IC95% 1,36–3,13).

**Conclusão:**

Observam-se iniquidades expressivas em saúde bucal entre beneficiários e não beneficiários do PBF. A superação destas exige integração entre políticas de transferência de renda, atenção primária e serviços odontológicos especializados, orientada pelos princípios da equidade e justiça social.

## INTRODUÇÃO

A saúde bucal é parte integral da saúde global e desempenha papel fundamental na alimentação, fala, autoestima e no bem-estar psicológico^1^. Problemas odontológicos como cárie, doença periodontal e perda dentária contribuem não apenas para dor, comprometimento funcional, mas também estão associados a uma pior qualidade de vida, dificuldades nutricionais e um risco aumentado de doenças sistêmicas — incluindo doenças cardiovasculares, diabetes, síndrome metabólica, infecções respiratórias e desfechos adversos na gravidez^1,2^.

As doenças bucais permanecem entre os agravos mais prevalentes em saúde pública e constituem importantes marcadores de iniquidades sociais^3^, diferenças ou desigualdades que são desnecessárias, evitáveis e eticamente injustas^4^. Estima-se que os problemas bucais e as necessidades protéticas afetem de forma mais intensa populações em situação de vulnerabilidade, ampliando disparidades regionais e socioeconômicas historicamente observadas no Brasil^3,5^.

Indivíduos de menor renda, menor escolaridade ou pertencentes a grupos vulneráveis apresentam maior prevalência e gravidade de cárie dentária, doença periodontal e perda dentária, e menor utilização de serviços odontológicos^2,6,7^. Além disso, revisões críticas e sistemáticas mostram que essas iniquidades são universais e persistentes ao longo do tempo, afetando crianças, adultos e idosos em diferentes países e sistemas de saúde^8–10^.

No Brasil, revisões sistemáticas e estudos de coorte de base populacional identificaram consistentemente uma forte associação entre menor status socioeconômico, medido por renda, educação e classe econômica, e piores desfechos de saúde bucal, incluindo maior prevalência de cárie não tratada, edentulismo e autoavaliação ruim da saúde bucal entre adultos e crianças brasileiros^11–13^.

Nesse cenário, iniciativas foram tomadas visando reduzir ou minimizar as desigualdades socioeconômicas e as de saúde bucal no país. O Programa Bolsa Família (PBF), programa de transferência de renda condicionada do governo federal do Brasil, foi implantado em 2003 com o objetivo de combater a fome e a pobreza no Brasil^14^. O Brasil Sorridente, instituído em 2004 pelo Ministério da Saúde, promoveu a incorporação da odontologia na atenção primária, a reposição de equipamentos, a formação de equipes, além de ações de promoção, prevenção e recuperação da saúde bucal^15^. Nesse sentido, poder-se-ia esperar que essas políticas contribuíssem de forma complementar para a redução de barreiras financeiras e para a ampliação do acesso equitativo aos serviços de saúde bucal no Brasil. Entretanto, tem sido argumentado que elas carecem de articulação entre si^16^. Apesar dos avanços alcançados com essas políticas públicas, as iniquidades em saúde bucal e no acesso aos serviços odontológicos^17^ ainda persistem, afetando de forma desproporcional os grupos mais vulneráveis. Crianças e escolares beneficiários apresentam maior prevalência e gravidade de cárie dentária, além de menor acesso a serviços odontológicos, em relação aos não beneficiários do PBF^18^.

Contudo, há escassez de estudos nacionais abrangentes, que comparem múltiplos indicadores de saúde bucal entre beneficiários e não beneficiários do PBF, em diferentes grupos etários. Nesse sentido, o objetivo do presente estudo é estimar as prevalências e comparar as diferenças nas condições de saúde bucal entre beneficiários e não beneficiários do Programa Bolsa Família em crianças, adolescentes, adultos e idosos, com base em dados representativos da população nacional.

## MÉTODOS

### Desenho do estudo e fonte de dados

Este estudo transversal utilizou dados da Pesquisa Nacional de Saúde Bucal (SB Brasil) 2023, levantamento domiciliar conduzido em todo o território nacional, cuja metodologia detalhada encontra-se descrita no relatório final da pesquisa^5^. O estudo foi projetado para estimar indicadores de saúde bucal por faixas etárias e 53 domínios geográficos (Brasil, Unidades Federativas, Distrito Federal e capitais estaduais). As variáveis clínicas foram coletadas por examinadores treinados e calibrados e registradas em sistema eletrônico, utilizando-se critérios e recomendações da Organização Mundial da Saúde (OMS).

### População e amostragem

A população incluiu residentes em domicílios urbanos e rurais do Brasil. A amostragem foi probabilística, complexa, por conglomerados em estágios múltiplos. Foram examinados indivíduos nas seguintes idades: 5 anos (dentição decídua); 12 anos (dentição permanente); 15–19 anos (adolescentes); 35–44 anos (adultos); e 65–74 anos (idosos), permitindo estimar indicadores comparáveis internacionalmente e avaliar o curso de vida da saúde bucal. Resultados para a idade de 5 anos constam em tabelas suplementares. O tamanho amostral foi calculado considerando prevalências esperadas de cárie e doença periodontal, precisão de 5%, efeito de desenho (deff=2) e acréscimo de 20% para perdas, totalizando cerca de 50 mil indivíduos em todas as regiões do país.

### Treinamento e calibração

Os examinadores foram cirurgiões-dentistas previamente treinados e submetidos à calibração teórico-prática. A concordância intra e interexaminadores foi avaliada por meio do coeficiente Kappa (mínimo aceitável ≥0,65), assegurando a padronização dos critérios diagnósticos para todas as condições bucais investigadas. Os anotadores também passaram por capacitação específica para garantir a fidedignidade dos registros.

### Coleta de dados e controle de qualidade

A coleta ocorreu em visitas domiciliares, sendo composta por: entrevista estruturada, aplicada a participantes ou responsáveis legais (no caso de menores), abrangendo dados sociodemográficos, comportamentais e de utilização de serviços odontológicos; além de exames clínicos realizados com espelhos planos e sondas da OMS, sob luz natural, em local arejado e iluminado do domicílio seguindo normas de biossegurança. Foram avaliadas as seguintes condições: cárie dentária (índices ceo-d/CPO-D), consequências clínicas da cárie (PUFA), necessidade de tratamento, saúde periodontal (CPI), edentulismo, uso e necessidade de prótese, má oclusão (DAI) e traumatismo dentário.

### Variáveis

#### Desfechos

Foram analisados indicadores clínicos e subjetivos de saúde bucal nas faixas etárias investigadas (crianças, adolescentes, adultos e idosos). As variáveis subjetivas incluíram: dor de dente nos últimos seis meses (sim/não); urgência percebida no tratamento odontológico (sem necessidade; preventivo ou de rotina; tratamento eletivo; necessidade de tratamento imediato); e autopercepção da saúde bucal (regular/boa/muito boa; ruim/muito ruim). A decisão de combinar as categorias "regular", "muito boa" e "boa" é consistente com a literatura epidemiológica e teve como objetivo distinguir indivíduos que percebem nitidamente sua saúde bucal como problemática daqueles que não relatam problemas relevantes, uma vez que a categoria "regular" tende a representar uma avaliação intermediária ou neutra, enquanto "ruim" e "muito ruim" indicam explicitamente a presença de problemas ou insatisfação com a saúde bucal.

As variáveis clínicas compreenderam a avaliação da cárie dentária, mensurada pelos índices ceo-d (dentição decídua) e CPO-D (dentição permanente), incluindo o número de dentes cariados, restaurados e perdidos. A presença de cárie não tratada foi categorizada em "nenhum" ou "um ou mais dentes". As consequências clínicas da cárie foram mensuradas pelo índice PUFA (PUFA=0; PUFA≥1). Nos adultos e idosos, também foram avaliadas a presença de cárie radicular (sim/não) e a ocorrência de dentes permanentemente extraídos (sim/não).

A necessidade de tratamento odontológico foi classificada em restauração, tratamento pulpar ou extração (nenhum/≥1 dente nas faixas mais jovens; sim/não nos adultos e idosos). Entre adultos e idosos, também foram incluídas as condições periodontais, avaliadas pela presença de alguma perda de inserção periodontal e de bolsas periodontais (sim; não). O edentulismo foi definido como a ausência total de dentes naturais (dentado; edêntulo), e a necessidade de prótese dentária foi categorizada em não necessita, necessita prótese parcial ou necessita prótese total. Com exceção de algumas categorizações, foram utilizados os indicadores já calculados no banco de dados disponibilizado pelo Ministério da Saúde.

#### Exposição principal

A exposição principal foi avaliada por uma questão inquirindo se algum morador havia recebido rendimento proveniente de benefício assistencial do Programa Bolsa Família no último ano (não; sim). Foram excluídos participantes que não sabiam ou não responderam a essa questão supracitada.

#### Outras variáveis independentes

As variáveis independentes incluíram características sociodemográficas, contextuais e de uso de serviços odontológicos. As variáveis contextuais abrangeram região geográfica do Brasil (Norte; Nordeste; Sudeste; Sul; Centro-Oeste) e tipo de cidade (capital; interior). As características individuais compreenderam sexo (masculino; feminino) e raça/cor da pele (branca; preta; amarela; parda; indígena).

A condição socioeconômica foi avaliada por meio de renda familiar mensal, renda per capita e número de pessoas por cômodo do domicílio (todas em valores contínuos); além da escolaridade da mãe e da escolaridade do participante, classificadas em oito categorias: não estuda, fez Educação de Jovens e Adultos (EJA), Ensino Fundamental (EF) incompleto/completo, Ensino Médio (EM) incompleto/completo e Ensino Superior (ES) incompleto/completo.

O uso de serviços odontológicos foi analisado segundo a última consulta ao dentista (nunca; até 1 ano; mais de 1 a 2 anos; mais de 2 a 3 anos; mais de 3 anos) e o principal motivo da consulta (nunca foi ao dentista; limpeza, prevenção ou revisão; dor de dente; extração; tratamento dentário; problema gengival; tratamento de ferida bucal; implante dentário; colocação/manutenção de aparelho ortodôntico; colocação/manutenção de prótese ou dentadura; outros).

### Entrada e análise dos dados

Os dados foram duplamente digitados com validação automática para garantir consistência e qualidade. As análises consideraram o delineamento amostral complexo, incorporando pesos, estratos e conglomerados, conforme especificações técnicas do SB Brasil 2023 e com o comando *svy* no software *Stata* 15.1.

Inicialmente, foram calculadas frequências, médias e prevalências dos principais problemas bucais, estratificadas segundo a condição de beneficiários ou não do PBF e grupos etários pesquisados. Em seguida, estimaram-se as Razões de Prevalências (RP) ajustadas para cada desfecho específico de saúde bucal por meio de modelos de regressão de Poisson múltipla e testes de Wald para tendência linear e para heterogeneidade de proporções, tendo como variável de exposição principal o recebimento do benefício. Para o desfecho necessidade de prótese, entre adultos e idosos, foram estimadas as Razões de Chances (OR) ajustadas por meio de regressão logística multinomial múltipla, uma vez que se trata de um desfecho categórico nominal com mais de duas categorias sem ordenamento natural. Os modelos foram ajustados para potenciais fatores de confusão, identificados com base na literatura e significância estatística (p<0,05). Para evitar colinearidade com a exposição principal, variáveis socioeconômicas não foram incluídas nos modelos multivariados. Assim, o efeito de ser beneficiário do PBF foi ajustado por sexo, cor da pele e realização de consulta odontológica nos últimos 12 meses.

### Aspectos éticos

O estudo foi aprovado pela Comissão Nacional de Ética em Pesquisa (Conep – Comissão Nacional de Ética em Pesquisa/CNS – Conselho Nacional de Saúde/MS – Ministério da Saúde, CAAE 34497120.6.3001.0008, parecer número 4.823.054), e todos os participantes assinaram o Termo de Consentimento Livre e Esclarecido (TCLE) ou, no caso de menores, o Termo de Assentimento e consentimento dos responsáveis. A pesquisa seguiu os princípios éticos da Declaração de Helsinque.

#### Declaração de disponibilidade de dados:

Todo o conjunto de dados que dá suporte aos resultados deste estudo está disponível mediante solicitação ao autor correspondente. O conjunto de dados não está publicamente disponível devido à integração de bancos de dados realizados pelos autores.

## RESULTADOS

Ao todo, 40.720 indivíduos participaram do SB Brasil 2023. Destes, 3.200 não souberam informar ou não responderam se haviam recebido rendimentos provenientes do Programa Bolsa Família e, portanto, foram excluídos das análises. Assim, as análises foram conduzidas com 37.520 participantes, distribuídos entre os diferentes grupos etários. Do total analisado, 11.757 (31,4%) eram beneficiários do PBF, e 25.763 (68,6%) pertenciam à população geral não beneficiária.

Beneficiários do referido programa apresentaram perfil distinto em relação à população geral. Houve maior concentração nas regiões Nordeste e Sudeste, enquanto na população geral predominaram o Sudeste e o Nordeste. Do ponto de vista socioeconômico, como esperado, os beneficiários exibiram piores condições de renda e escolaridade, com renda familiar e per capita significativamente menores e maior proporção de participantes com ensino fundamental incompleto. Em relação à utilização de serviços odontológicos, os beneficiários do PBF também o fizeram em sua maioria para realizar limpeza, prevenção ou revisão, entretanto, a proporção de uso por motivo de dor de dente entre os beneficiários foi maior do que na população geral (Tabela 1).

**Tabela 1:** Características demográficas, socioeconômicas e de utilização de serviços odontológicos segundo condição de beneficiário do Programa Bolsa Família. Pesquisa Nacional de Saúde Bucal (SB Brasil), 2023.

Variáveis		Bolsa Família			População geral		
		n	%	IC95%	n	%	IC95%
Regiões do Brasil							
	Norte	3.814	15,8	12,7–19,6	5.523	6,4	5,1–8,0
	Nordeste	5.294	44,4	39,0–49,9	8.216	20,6	17,6–23,9
	Sudeste	791	25,1	20,6–30,3	3.712	47,4	41,2–53,7
	Sul	697	6,6	4,9–8,7	4.362	17,4	14,6–20,7
	Centro-Oeste	1.161	7,9	5,1–12,0	3.950	7,9	6,0–10,4
Tipo de cidade							
	Capital	8.452	22,7	19,9–25,7	18.914	23,0	20,0–26,4
	Interior	3.305	77,3	74,2–80,1	6.849	76,9	73,5–79,9
Faixa etária (anos)							
	5	3.051	7,29	6,5–8,2	3.433	2,89	2,5–3,4
	12	2.487	6,5	5,7–7,4	3.499	3,3	2,8–3,8
	15–19	2.583	30,7	28,1–33,5	4.791	18,6	16,7–20,6
	35–44	2.481	47,7	44,6–50,9	6.006	50,0	47,2–52,8
	65–74	1.155	7,6	6,5–8,9	8.034	25,1	23,2–27,0
Sexo							
	Masculino	3.896	35,7	33,1–38,3	10.539	40,9	39,2–42,5
	Feminino	7.861	64,3	61,6–66,8	15.223	59,0	57,4–60,7
Raça/cor							
	Branca	2.556	24,4	21,3–27,8	12.511	48,7	44,9–53,2
	Preta	1.692	17,3	13,7–21,5	2.972	11,0	9,9–12,3
	Amarela	165	1,6	1,0–2,7	283	1,0	0,7–1,4
	Parda	7.150	55,7	50,7–58,9	8.764	38,0	34,7–42,3
	Indígena	58	0,6	0,3–1,2	80	0,3	0,1–0,5
	Não soube informar	136	1,3	0,7–2,4	1.153	1,2	0,7–1,9
Renda familiar							
		8.727	1.626*	1.502–1.749	18.907	3569,1*	3.310–3.828
Renda per capita							
		8.705	422,2*	389–456	18.879	1.197*	1.103–1.290
Pessoas/cômodo							
		11.656	1,9*	1,9–2,1	25.539	1,4*	1,3–1,5
Escolaridade do participante							
	Sem instrução	298	2,8	2,1–3,9	1.045	3,5	2,8–4,2
	Fundamental incompleto	4.112	32,6	29,2–36,3	7.792	22,0	19,8–24,4
	Fundamental completo/Médio incompleto	2.321	34,7	31,7–37,8	4.992	24,9	22,4–27,6
	Médio completo ou mais	1.865	28,6	25,4–32,0	8.240	48,1	45,2–51,0
	Não respondeu	110	1,3	0,7–2,3	261	1,5	0,8–2,7
Última consulta ao dentista							
	Nunca	2.147	7,6	6,1–9,4	1.838	2,3	1,8–2,8
	Até um ano	4.771	43,7	39,9–47,7	12.389	48,1	45,0–51,2
	Mais de 1 ano a 2 anos	1.924	18,6	16,0–21,7	4.436	17,6	16,1–19,1
	Mais de 2 anos a 3 anos	1.036	9,8	8,3–11,7	2.630	11,6	10,2–13,2
	Mais de 3 anos	1.374	14,7	11,9–18,1	3.955	15,7	13,8–17,8
	Não sabe	505	5,4	3,6–7,9	1.141	4,7	3,1–7,0
Principal motivo da consulta							
	Nunca foi ao dentista	2.147	7,5	6,1–9,4	1.838	2,3	1,8–2,8
	Limpeza, prevenção ou revisão	3.318	25,9	23,5–28,6	826	30,7	27,8–33,5
	Dor de dente	1.811	17,3	15,2–19,8	2.763	12,9	10,9–15,0
	Extração	1.303	13,2	11,4–15,5	2.761	8,9	7,7–10,1
	Tratamento dentário	1.377	16,3	13,8–19,3	3.501	17,5	15,7–19,4
	Problema de gengiva	58	0,5	0,3–1,0	209	0,8	0,5–1,1
	Ferida na boca	18	0,1	0,2–0,1	55	0,2	0,1–0,5
	Implante dentário	13	0,1	0,0–0,3	324	1,8	1,2–2,7
	Consulta ortodontia	461	6,2	5,0–7,8	1.434	6,6	5,5–7,7
	Consulta prótese	382	3,6	2,8–4,7	2.599	9,9	8,6–12,1
	Outros	207	2,2	1,5–3,4	521	2,6	1,9–3,4

* Médias. IC: intervalo de confiança.

Entre as crianças e os adolescentes participantes, observaram-se diferenças significativas nas condições de saúde bucal entre beneficiários e não beneficiários. Em todas as faixas etárias analisadas (12 e 15–19 anos), os beneficiários apresentaram maior prevalência de dor de dente, pior autopercepção de saúde bucal e maiores necessidades de tratamento odontológico, especialmente de caráter imediato. Além disso, os índices de experiência de cárie (CPOD) e de complicações decorrentes da cárie (PUFA) foram consistentemente mais elevados entre beneficiários em relação aos não beneficiários (Tabela 2).

**Tabela 2:** Condições de saúde bucal em crianças e adolescentes de acordo com a condição de beneficiário do Programa Bolsa Família. Pesquisa Nacional de Saúde Bucal (SB Brasil) 2023.

Variáveis		12 anos						15 a 19 anos					
		Bolsa Família			População geral			Bolsa Família			População geral		
		n	%	IC95%	n	%	IC95%	n	%	IC95%	n	%	IC95%
Dor dentária													
	Não	1.911	77,7	73,3–81,5	2.926	85,9	82,5–88,7	1.926	74,7	70,4–8,6	3.820	80,5	77,5–83,3
	Sim	563	22,4	18,5–26,7	554	14,1	11,3–17,4	642	25,3	21,4–29,5	945	19,5	16,7–2,52
Autopercepção em saúde bucal													
	Boa/muito boa	2.263	93,7	91,3–95,5	3.297	97,2	96,1–97,9	2.354	91,1	87,5–93,7	4.509	96,4	94,4–97,7
	Regular/ruim/muito ruim	171	6,3	4,5–8,7	144	2,8	2,0–3,8	193	8,9	6,2–12,5	218	3,6	2,2–5,5
Necessidade de urgência													
	Sem necessidade	455	22,6	18,6–27,1	997	37,1	32,4–41,9	451	19,3	15,1–24,1	1.319	34,1	26,9–41,9
	Tratamento preventivo	840	31,9	27,9–36,0	1.128	27,7	24,6–30,9	774	27,7	22,6–33,2	1.382	28,7	24,2–33,5
	Tratamento eletivo	943	37,4	33,2–41,7	1.172	30,8	25,6–36,4	1.031	39,2	33,6–45,1	1.709	31,8	27,7–36,2
	Necessidade de tratamento imediato	245	8,2	6,0–10,9	201	4,4	3,2–5,9	323	13,9	10,6–17,8	373	5,4	4,1–7,1
CPO-D*													
		2.483	1,8	1,6–2,1	3.498	1,5	1,2–1,7	2.579	3,7	3,2–4,2	4.783	3,1	2,6–3,5
Cariados*													
		2.483	1,3	1,1–1,4	3.498	0,9	0,7–1,0	2.579	2,2	1,8–2,5	4.783	1,2	0,9–1,4
Restaurados*													
		2.483	0,5	0,3–0,6	3.498	0,6	0,4–0,7	2.579	1,0	0,7–1,2	4.783	1,5	1,2–1,7
Perdidos*													
		2.483	0,1	0,05–0,1	3.498	0,1	0,03–0,07	2.579	0,6	0,3–0,7	4.783	0,5	0,3–0,6
Cárie não tratada													
	C=0	1.350	57,2	53,2–61,1	2.327	68,3	62,5–73,5	1.284	44,8	38,9–50,8	2.946	62,5	58,1–66,7
	C≥1	1.133	42,8	38,8–46,8	1.171	31,7	26,4–37,4	1.295	55,2	49,2–61,1	1.837	37,5	33,2–41,9
Experiência de cáries													
	CPO-D = 0	1.043	46,2	41,9–50,4	1.833	53,9	48,3–59,4	819	28,5	23,6–33,8	1.776	36,9	32,4–41,6
	CPO-D≥1	1.440	53,9	49,5–58,1	1.665	46,1	40,5–51,6	1.760	71,5	66,2–76,3	3.007	63,1	58,3–67,5
Prevalência PUFA													
	PUFA=0	2.176	88,1	85,0–90,6	3.222	93,9	92,1–95,3	2.121	79,6	73,4–84,7	4.258	91,4	88,9–93,2
	PUFA≥1	307	11,9	9,3–14,9	276	6,1	4,7–7,8	458	20,4	15,3–26,6	525	8,6	6,7–11,0
Necessidade de tratamento													
	Nenhum dente	1.382	58,6	54,5–62,4	2.356	70,3	65,9–74,3	1.303	47,5	42,1–53,0	2.961	63,4	58,4–68,1
	≥1 dente	1.101	41,4	37,5–45,4	1.142	29,7	25,6–34,1	1.276	52,5	46,9–57,9	1.822	36,6	31,8–41,5
Necessidade de restauração													
	Nenhum	1.473	62,2	58,2–66,0	2.437	71,7	67,4–75,7	1.412	52,0	46,6–57,3	3.126	66,1	61,4–70,5
	≥1 dente	1.010	37,8	33,9–41,8	1.061	28,3	24,2–32,6	1.167	48,0	42,6–53,3	1.657	33,9	29,4–38,5
Necessidade de tratamento pulpar													
	Nenhum dente	2.262	91,8	89,1–93,8	3.305	95,5	94,0–96,6	2.289	88,2	84,8–90,9	4.438	94,3	92,7–95,6
	≥1 dente	221	8,2	6,15–10,9	193	4,5	3,3–5,9	290	11,8	9,0–15,1	345	5,7	4,3–7,3
Necessidade de extração													
	Nenhum dente	2.374	96,0	94,3–97,2	3.411	98,4	97,6–98,9	2.339	88,6	84,7–91,5	4.508	95,8	94,3–96,9
	≥1 dente	109	4,0	2,7–5,7	87	1,6	1,0–2,3	240	11,4	8,5–15,2	275	4,2	3,1–5,7
Prevalência extraído													
	E=0	2.320	93,9	92,0–95,3	3.355	96,6	95,2–97,5	2.110	80,9	76,8–84,3	4.207	89,4	86,9–91,3
	E≥1	163	6,1	4,6–7,9	143	3,4	2,4–4,7	469	19,1	15,7–23,1	576	10,7	8,6–13,0

* Médias. IC: intervalo de confiança.

No que se refere ao traumatismo dentário, às oclusopatias e à presença de sangramento e cálculo, não foram observadas diferenças expressivas entre beneficiários e não beneficiários do Bolsa Família (dados não apresentados).

Com relação aos adultos e idosos, observou-se um padrão consistente de piores condições de saúde bucal entre aqueles que eram beneficiários em comparação aos que não eram. Em ambos os grupos, os beneficiários apresentaram pior autopercepção de saúde bucal, maior ocorrência de dor dentária e maiores necessidades de tratamento, especialmente de caráter imediato. Além disso, verificou-se maior experiência e gravidade de cárie, com predominância de perdas dentárias e menor proporção de dentes restaurados entre os beneficiários. Destaca-se, ainda, que a demanda de tratamento reabilitador, como a necessidade de próteses parciais ou totais, foi aproximadamente duas vezes mais frequente nos beneficiários do que na população geral (Tabela 3).

**Tabela 3:** Condições de saúde bucal em adultos e idosos de acordo com condição de beneficiário do Programa Bolsa Família. Pesquisa Nacional de Saúde Bucal (SB Brasil) 2023.

Variáveis		35 a 44 anos						65 a 74 anos					
		Bolsa Família			População geral			Bolsa Família			População geral		
		n	%	IC95%	n	%	IC95%	n	%	IC95%	n	%	IC95%
Dor dentária													
	Não	1.654	70,4	65,2–75,2	4.603	77,4	73,8–80,7	928	89,4	84,9–92,6	6.676	87,7	85,2–89,8
	Sim	811	29,5	24,7–34,8	1.347	22,5	19,2–26,2	149	10,6	7,3–15,0	956	12,3	10,2–14,7
Autopercepção em saúde bucal													
	Boa/muito boa	1.857	75,3	70,4–79,7	5.146	87,3	84,7–89,6	856	74,6	66,8–81,1	6.476	86,48	84,4–88,3
	Regular/ruim/muito ruim	592	24,6	20,2–29,5	767	12,6	10,3–15,2	247	25,4	18,9–33,2	1263	13,52	11,6–15,6
Necessidade de urgência													
	Sem necessidade	242	12,4	9,3–16,3	1.242	21,3	17,1–26,1	265	28,2	21,5–36,1	2.045	29,1	25,2–33,3
	Tratamento preventivo	393	19,9	15,3–25,5	1.242	22,0	18,8–25,5	155	9,7	7,0–13,3	1.270	16,9	14,4–19,9
	Tratamento eletivo	1.365	51,6	46,3–56,9	2.896	48,2	42,9–53,5	578	52,6	44,8–60,3	4.044	46,4	42,2–50,6
	Necessidade de tratamento imediato	475	15,9	12,7–19,9	600	8,4	6,6–10,6	156	9,3	6,2–13,8	654	7,4	5,8–9,4
CPO-D*													
		2.475	11,8	10,8–12,8	5.980	10,5	9,9–11,1	1.154	25,2	23,8–26,5	8.014	23,3	22,6–24,0
Cariados*													
		2.475	2,9	2,4–3,4	5.980	1,6	1,4–1,8	1.154	0,9	0,7–1,2	8.014	0,9	0,7–1,1
Restaurados*													
		2.475	3,9	3,4–4,4	5.980	5,9	5,5–6,3	1.154	1,2	0,9–1,5	8.014	3,0	2,4–3,6
Perdidos*													
		2.475	4,9	4,2–5,7	5.980	3,0	2,6–3,5	1.154	22,9	21,4–24,5	8.014	19,3	18,2–20,4
Prevalência PUFA													
	PUFA=0	1.755	75	70,3–79,1	4.939	83,4	80,6–85,9	982	89,1	85,7–91,8	7.137	90,0	88,1–91,6
	PUFA≥1	720	25	20,8–29,6	1.041	16,6	14,1–19,4	172	10,8	8,1–14,2	878	9,9	8,3–11,8
Cárie raiz													
	Nenhum dente	1.773	70,2	64,5–75,3	4.922	82,3	78,5–85,5	924	81,4	75,1–86,4	6.662	83,0	79,7–85,9
	≥1 dente	702	29,8	24,6–35,5	1.058	17,7	14,5–21,4	230	18,5	13,5–24,8	1.353	16,96	14,08–20,3
Necessidade de tratamento													
	Nenhum dente	769	32,1	26,5–38,2	2.986	49,7	45,5–53,8	755	67,0	59,6–73,8	5.424	69,2	65,5–72,6
	≥1 dente	1.706	67,8	61,8–73,4	2.994	50,3	46,1–54,4	399	32,9	26,2–40,4	2.591	30,7	27,3–34,4
Necessidade de restauração													
	Nenhum dente	948	37,7	31,9–43,8	3.349	55,8	52,1–59,5	845	75,6	69,2–81,0	5.962	74,1	70,5–77,4
	≥1 dente	1.527	62,3	56,1–68,0	2.631	44,1	40,5–47,9	309	24,3	18,9–30,7	2.053	25,9	22,5–29,5
Necessidade de tratamento pulpar													
	Nenhum dente	2.166	88,3	84,9–91,0	5.497	91,5	89,1–93,4	1.110	96,7	94,6–98,0	7.755	96,8	95,3–97,9
	≥1 dente	309	11,7	8,9–15,1	483	8,5	6,5–10,8	44	3,2	1,9–5,4	260	3,1	2,1–4,6
Necessidade de extração													
	Nenhum dente	1.875	79,3	75,4–82,7	5.215	87,9	85,4–90,1	989	87,9	81,9–92,1	7.098	90,1	88,2–91,7
	≥1 dente	600	20,6	17,3–24,5	765	12,0	9,9–14,5	165	12,1	7,87–18,1	917	9,8	8,27–11,7
Extraído													
	Nenhum dente	748	28,9	23,3–35,3	2.623	43,3	38,0–48,8	78	2,7	1,6–4,5	732	10,8	8,6–13,5
	≥1 dente	1.727	71,0	64,7–76,6	3.357	56,6	51,2–61,9	1.076	97,2	95,5–98,3	7.282	89,2	86,4–91,4
Perda inserção													
	Não	1.541	83,7	78,8–87,6	4.306	84,3	78,7–88,7	83	69,9	54,5–81,8	773	59,6	49,7–68,8
	Sim	289	16,3	12,3–21,2	674	15,6	11,3–21,2	41	30,1	18,1–45,4	481	40,3	31,1–50,2
Bolsa													
	Não	1.529	83,8	78,3–88,1	4.372	85,8	82,4–88, 6	93	76,3	61,0–86,9	998	77,2	69,9–83,2
	Sim	301	16,1	11,8–21,6	608	14,1	11,3–17,6	31	23,6	13,0–38,9	258	22,7	16,7–30,1
Edentulismo													
	Não	2.434	98,6	96,4–99,4	5.912	99,3	98,8–99,6	683	53,6	45,8–61,3	5.270	65,4	61,9–68,7
	Sim	41	1,3	0,5–3,5	68	0,6	0,3–1,1	471	46,3	38,7–54,1	2.744	34,5	31,2–38,0
Necessidade de prótese													
	Não necessita	743	30,4	25,1–36,2	2.967	50,8	45,0–56,5	198	13,0	9,7–17,3	1.947	26,2	23,1–29,6
	Necessita parcial	1.622	65,3	59,4–70,7	2.877	47,0	41,1–53,0	393	31,6	25,8–38,0	2.875	36,3	33,6–39,1
	Necessita total	108	4,3	2,7–6,8	135	2,1	1,3–3,4	562	55,3	48,7–61,8	3.190	37,4	33,9–41,1

* Médias.

IC: intervalo de confiança.

Após ajuste por sexo, cor da pele e consulta odontológica, observou-se que crianças e adolescentes beneficiários do Programa Bolsa Família apresentaram maiores prevalências de dor dentária, autopercepção negativa da saúde bucal e necessidade de tratamento odontológico quando comparados aos não beneficiários. As razões de prevalência ajustadas indicaram associação significativa entre o recebimento do benefício e maior ocorrência de cárie não tratada, complicações decorrentes da cárie (PUFA) e necessidade de tratamento imediato (Tabela 4).

**Tabela 4:** Associação entre condição de beneficiário do Programa Bolsa Família (versus não beneficiário) em diferentes desfechos de saúde bucal e de acordo com a idade. Pesquisa Nacional de Saúde Bucal (SB Brasil) 2023.

Variável	12 anos		15 a 19 anos		35 a 44 anos		65 a 74 anos	
	RP	IC95%	RP	IC95%	RP	IC95%	RP	IC95%
Autopercepção saúde bucal	1,87	1,19–2,94	2,11	1,12–3,97	1,84	1,48–2,28	1,67	1,25–2,22
Dor de dente	**1,47**	**1,10–1,95**	1,21	0,98–1,50	**1,29**	**1,08–1,55**	0,96	0,65–1,43
CPOD	1,15	0,94–1,41	1,11	0,96–1,31	**1,11**	**1,02–1,21**	1,02	0,73–1,46
Cariado	1,28	1,04–1,59	**1,64**	**1,34–2,01**	**1,77**	**1,50–2,09**	1,08	1,00 –1,17
Perdido	1,22	0,75–1,99	1,15	0,67–1,97	**1,54**	**1,25–1,90**	**1,06**	**1,03–1,09**
Restaurado	0,92	0,67–1,25	**0,66**	**0,50–0,86**	**0,69**	**0,59–0,80**	0,60	0,43–0,83
Cariado ≥1	**1,22**	**1,01–1,47**	**1,35**	**1,22–1,50**	**1,35**	**1,22–1,49**	1,09	0,85–1,40
Extraído ≥1	1,41	0,95–2,09	**1,63**	**1,19–2,24**	**1,19**	**1,07–1,32**	1,06	1,03–1,09
PUFA – permanentes	**1,67**	**1,16–2,38**	**2,03**	**1,45–2,83**	**1,49**	**1,18–1,89**	1,01	0,72–1,40
Necessidade de tratamento	**1,28**	**1,10–1,50**	**1,32**	**1,14–1,53**	**1,31**	**1,19–1,45**	1,06	0,82–1,36
Necessidade de restauração	**1,24**	**1,06–1,46**	**1,31**	**1,12–1,53**	**1,38**	**1,24–1,54**	0,95	0,72–1,25
Necessidade de endodontia	**1,58**	**1,03–2,43**	**1,81**	**1,29–2,54**	1,35	0,95–1,91	0,95	0,47–1,89
Necessidade de extração	2,32	**1,24–4,33**	**2,11**	**1,40–3,18**	**1,79**	**1,40–2,29**	1,05	0,66–1,70
Necessidade de urgência	**1,65**	**1,05–2,56**	**2,29**	**1,54–3,39**	**1,91**	**1,42–2,57**	1,05	0,65–1,73
Necessidade de prótese parcial*			2.66	1,65–4,29	2,11	1,56–2,85	1,61	1,05–2,47
Necessidade de prótese total*			6,64	0,48–91,8	**3,38**	**1,65–3,42**	**2,06**	**1,36–3,13**
Cárie raiz ≥1					**1,61**	**1,26–2,06**	1,08	0,75–1,58
Perda de inserção					1,01	0,66–1,54	0,70	0,44–1,10
Bolsa periodontal					1,16	0,79–1,71	0,89	0,50–1,60
Edentulismo					2,28	0,71–7,31	1,16	0,97–1,40

Nota: Valores em negrito são estatisticamente significativos (p<0,005).

* Razão de Chances (OR). Ajustado para sexo, cor da pele e consulta odontológica nos últimos 12 meses.

RP: razão de prevalência; IC: intervalo de confiança.

Entre os adultos, o padrão observado foi semelhante ao das faixas etárias mais jovens na análise ajustada, indicando a persistência das iniquidades em saúde bucal. Os beneficiários do PBF apresentaram maiores prevalências de autopercepção ruim de saúde bucal, dor, piores indicadores clínicos e maior necessidade de tratamento quando comparados aos não beneficiários. No que se refere à reabilitação protética, chama a atenção o fato de que a necessidade de próteses totais e parciais foi notadamente maior entre os beneficiários em comparação aos não beneficiários, tanto entre adultos quanto entre idosos (Tabela 4).

## DISCUSSÃO

Os resultados deste estudo evidenciam iniquidades marcantes em saúde bucal entre beneficiários e não beneficiários do Programa Bolsa Família em todas as faixas etárias analisadas. Iniquidades em saúde bucal referem-se às desigualdades ou diferenças injustas entre diferentes grupos populacionais pertinentes a saúde oral e acesso a serviços odontológicos, relacionando-se a fatores raciais, sociais, econômicos e territoriais^19,20^. Crianças, adolescentes, adultos e idosos vinculados ao programa apresentaram piores indicadores clínicos e subjetivos, maior prevalência de dor dentária, pior autopercepção de saúde bucal, maior experiência e gravidade de cárie, bem como maior necessidade de tratamento odontológico, sobretudo de caráter imediato^21^.

Essas iniquidades refletem um padrão amplamente descrito na literatura, segundo o qual a posição socioeconômica, expressa pela renda, escolaridade, ocupação e pelo local de residência, está fortemente associada a condições desfavoráveis de saúde bucal^6,7^. Revisões sistemáticas demonstram que indivíduos em situação de pobreza apresentam maiores taxas de cárie, perda dentária, periodontite e menor utilização de serviços odontológicos. Essas iniquidades resultam de determinantes sociais, incluindo acesso limitado a cuidados, baixa literacia em saúde, e barreiras culturais e geográficas ao cuidado odontológico^22–24^.

Embora as doenças bucais sejam amplamente preveníveis, elas permanecem em níveis alarmantes mundialmente por refletirem desigualdades estruturais, configurando uma "injustiça oral" global^2^.

Mesmo em países com sistemas universais de saúde, como o Brasil, as iniquidades persistem. Estudos nacionais mostram que, apesar da ampliação da atenção primária e da oferta gratuita de serviços odontológicos pelo Sistema Único de Saúde (SUS), os indivíduos de menor renda e escolaridade continuam utilizando menos os serviços preventivos e reabilitadores, recorrendo ao atendimento predominantemente em situações de dor ou urgência. Essa "inércia de iniquidade" reflete a reprodução de desvantagens históricas e barreiras geográficas e simbólicas ainda não superadas^21,22^.

O PBF, enquanto política de transferência condicionada de renda, é um instrumento consolidado de mitigação da pobreza. Evidências mostram seus efeitos positivos sobre desfechos como redução da mortalidade infantil e do baixo peso ao nascer. Contudo, seu impacto direto sobre os determinantes intermediários da saúde bucal e o acesso aos serviços odontológicos ainda é limitado^24,25^. Persistem obstáculos estruturais como distâncias das unidades de saúde, escassez de equipes em áreas vulneráveis e barreiras socioeconômicas que limitam o uso regular de serviços odontológicos e favorecem a busca apenas por urgências^26^.

Estudos nacionais e revisões sistemáticas^18,27,28^ apontam maior prevalência de cárie e menor uso de serviços odontológicos entre beneficiários de programas de transferência de renda em relação à população geral, indicando que a transferência de renda, embora essencial para reduzir vulnerabilidades econômicas, não é suficiente para eliminar barreiras estruturais nem garantir o acesso equitativo. Evidencia-se, portanto, a necessidade de integrar políticas de transferência de renda como o Bolsa Família com as Unidades Básicas de Saúde e as equipes de saúde bucal da Estratégia Saúde da Família. Medidas como incentivos à visita preventiva, priorização do atendimento odontológico para beneficiários e ações intersetoriais envolvendo saúde, educação, saneamento e assistência social. Isso converge com recomendações internacionais de políticas de combate à pobreza e saúde bucal^2,29^.

É provável que o Bolsa Família exerça benefícios sobre a saúde bucal entre os mais vulneráveis, sobretudo quando recebido por períodos prolongados. Um estudo em Fortaleza demonstrou redução de 87% na probabilidade de cárie em crianças beneficiárias por pelo menos dois anos^28^, sugerindo efeito protetor decorrente da melhoria socioeconômica. No entanto, quando a comparação se estende à população geral, as desigualdades permanecem evidentes, indicando que o programa contribui para atenuar iniquidades extremas, mas seu alcance é insuficiente para eliminar os efeitos estruturais da pobreza sobre desfechos em saúde.

Entre adultos e idosos, as maiores prevalências de dor, perda dentária e necessidade de prótese refletem o conceito de "desvantagem acumulada", em que a vulnerabilidade social limita tratamentos restauradores e perpetua práticas mutiladoras^2^.

Com exceção da necessidade de próteses totais e parciais, observou-se aparente menor disparidade entre beneficiários e não beneficiários nos demais indicadores de saúde bucal em idosos. Esse achado pode ser explicado pela elevada prevalência de edentulismo em ambos os grupos, que tende a reduzir as diferenças relativas. Tal padrão reflete o legado histórico de um modelo de atenção em saúde bucal predominantemente mutilador, anterior à implementação do Brasil Sorridente, que contribuiu para perdas dentárias generalizadas em diferentes estratos sociais^30^.

A maior perda dentária e consequente necessidade de próteses entre adultos e idosos beneficiários do PBF revelam barreiras no acesso ao atendimento reabilitador e de urgência e, ainda, acúmulo de desvantagens ao longo da vida. Nesse sentido, torna-se essencial fortalecer a integração do programa com os Centros de Especialidades Odontológicas (CEOs) do SUS.

A condição de beneficiário do PBF manteve-se associada a piores desfechos de saúde bucal, independentemente das variáveis de ajuste incluídas nos modelos^31,32^. Apesar de o Programa Bolsa Família contribuir para a redução da insegurança alimentar e melhoria das condições de vida, sua efetividade, especialmente em relação à saúde bucal, depende da articulação com políticas de saúde, especialmente com a atenção primária^26^. A persistência de um modelo de atenção centrado em procedimentos curativos limita o avanço da equidade e reforça a necessidade de integração entre proteção social e promoção da saúde bucal^22,33,34^.

A vigilância em saúde bucal constitui eixo estratégico para o monitoramento das iniquidades e a avaliação do impacto das políticas públicas. Sua articulação com a qualificação e expansão das equipes de saúde bucal e dos centros de especialidades representa alternativa concreta para a redução dessas iniquidades, uma vez que a presença dessas equipes está associada ao aumento de procedimentos preventivos, restauradores e reabilitadores^33^.

Este estudo apresenta limitações inerentes ao delineamento transversal, impossibilitando inferências causais. Há também a possibilidade de viés residual decorrente da ausência de variáveis contextuais relevantes, como fluoretação da água e cobertura das equipes de saúde bucal da família, e de fatores individuais, como tempo de permanência no programa e hábitos comportamentais relacionados à saúde bucal. Além disso, não foi avaliada a relevância do PBF na comparação entre diferentes níveis de vulnerabilidade, especialmente entre os grupos em situação de extrema pobreza, o que poderia aprofundar a compreensão sobre seus efeitos na redução das iniquidades. Os achados devem ser interpretados à luz da possibilidade de confundimento residual, uma vez que variáveis socioeconômicas centrais, como renda e escolaridade, embora fortemente relacionadas à elegibilidade ao PBF, não foram incluídas nos modelos ajustados. Assim, os resultados expressam desigualdades associadas à condição de beneficiário do programa. Por outro lado, a ampla amostra nacional e a padronização metodológica na coleta dos dados conferem robustez e elevada validade externa aos achados.

Os resultados confirmam profundas iniquidades em saúde bucal entre beneficiários e não beneficiários do Programa Bolsa Família, evidenciando o efeito persistente da vulnerabilidade social sobre desfechos bucais. O enfrentamento dessas iniquidades requer articulação efetiva entre políticas de proteção social e de saúde, especialmente com a atenção primária e os serviços odontológicos especializados, orientando ações públicas pelo princípio da justiça social e pelo direito universal à saúde bucal.
